# MiR-214 regulate gastric cancer cell proliferation, migration and invasion by targeting PTEN

**DOI:** 10.1186/1475-2867-13-68

**Published:** 2013-07-08

**Authors:** Ting-Song Yang, Xiao-Hu Yang, Xu-Dong Wang, Yi-Ling Wang, Bo Zhou, Zhen-Shun Song

**Affiliations:** 1 Department of Hepato-Biliary-Pancreatic Surgery, Tenth Peoples’ Hospital, School of Medicine, Tongji University, 301 Middle Yanchang Road, Shanghai 200072, China

**Keywords:** miR-214, PTEN, Gastric cancer, Proliferation, Invasion

## Abstract

**Background:**

MicroRNAs are a class of small non-coding RNAs that play an important role in various human tumor initiation and progression by regulating gene expression negatively. The aim of this study was to investigate the effect of miR-214 on cell proliferation, migration and invasion, as well as the functional connection between miR-214 and PTEN in gastric cancer.

**Methods:**

miR-214 and PTEN expression was determined in gastric cancer and matched normal tissues, and human gastric cancer cell lines by quantitative real-time PCR. The roles of miR-214 in cell proliferation, migration and invasion were analyzed with anti-miR-214 transfected cells. In addition, the regulation of PTEN by miR-214 was evaluated by Western blotting and luciferase reporter assays.

**Results:**

miR-214 was noted to be highly overexpressed in gastric cancer tissues and cell lines using qRT-PCR. The expression level of miR-214 is significantly associated with clinical progression and poor prognosis according to the analysis of the clinicopathologic data. We also found that the miR-214 levels are inversely correlated with PTEN in tumor tissues. And PTEN expression level is also associated with metastasis and invasion of gastric cancer. In addition, knockdown of miR-214 could significantly inhibit proliferation, migration and invasion of gastric cancer cells. Moreover, we demonstrate that PTEN is regulated negatively by miR-214 through a miR-214 binding site within the 3’-UTR of PTEN at the posttranscriptional level in gastric cancer cells.

**Conclusions:**

These findings indicated that miR-214 regulated the proliferation, migration and invasion by targeting PTEN post-transcriptionally in gastric cancer. It may be a novel potential therapeutic agent for gastric cancer.

## Background

Gastric cancer is the second most common cause of cancer-related death worldwide. It has been estimated that approximately 1 million patients are newly diagnosed with gastric cancer worldwide each year, which accounts for nearly 10% of all cancer deaths and claims approximately 700,000 lives annually [1,2]. Gastric cancer is a complex genetic disease, previous studies have demonstrated that several genes, known as oncogenes or tumor suppressors, were related to the initiation and progression of human gastric cancer [3], but the common molecular mechanisms of gastric cancer remain to be elucidated.

MicroRNAs, a class of small non-coding 18–25 nt in length RNAs, have been identified that it aberrantly expressed in several human malignancies, and could negatively regulate target gene expression by binding to the 3’-untranslated region (3’-UTR) of mRNAs for translational repression or degradation [4,5]. In the recent years, mounting evidence suggest that microRNAs plays a essential roles in tumor cell biological processes, such as cell proliferation, differentiation, migration and invasion [6-9].

A recent study determined the relationship between miRNA expression and progression of gastric cancer, which showed that 22 microRNAs were up-regulated, and 13 were down-regulated in gastric cancer, including miR-214 [10]. However, the specific role and molecular mechanism of miR-214 in gastric cancer cells remains unknown. Thus, we investigated the relationship between expression level of miR-214 and clinic pathological feature and prognosis in gastric cancer, and further studied the possible function of miR-214 in the gastric cancer cell line. Our study results show that overexpression of miR-214 is significantly associated with metastasis and invasion and poor prognosis in gastric cancer, moreover, it could negatively regulates PTEN by binding to their 3’-UTR regions to affect gastric cancer cell proliferation, migration and invasion.

## Materials and methods

### Human tissue samples and cell lines

Human tumor tissue samples and adjacent noncancerous controls were obtained by surgical resection from 120 patients with gastric cancer, at Department of General Surgery, Tenth peoples’ hospital, School of Medicine, Tongji University, Shanghai, China. All samples were derived from patients who had not received adjuvant treatment including radiotherapy or chemotherapy prior to surgery. All samples were snap-frozen and stored in liquid nitrogen after collection. Written informed consents were obtained from all subjects, and the study was approved and supervised by the Ethics Committee of Shanghai Tongji university.

The human gastric cancer cell lines SGC-7901, BGC-823 and normal gastric mucosa epithelial cell lines GES-1 were purchased from the Shanghai Institute of Biochemistry and Cell Biology (Shanghai, China). Cells were maintained in RPMI1640 (Invitrogen, US) supplemented with 10% fetal bovine serum (Invitrogen, US). All cells were incubated at 37°C in a humidified chamber supplemented with 5% CO_2_.

### RNA extraction and quantitative PCR

Total RNA from tissue sample and cells were isolated using TRIzol reagent (Invitrogen, US). The relative levels of miR-214 were examined by the altered stem-loop RT-PCR with specific RT and PCR primers using U6 snRNA as control. The primers for miR-214 were: Forward primer: 5’-AGCATAATACAGCAGGCACAGAC-3’; Reverse primer: 5’-AAAGGTTGTTCTCCACTCTCTCAC-3’. The expression of PTEN mRNA were detected by quantitative PCR using paired primers. β-actin gene was used as control. The primers for PTEN mRNA were: Forward primer: 5’-ACCAGTGGCACTGTT GTTTCAC-3’; Reverse primer: 5’-TTCCTCTGGTCCTGGTATGAAG-3’.

Quantitative PCR was performed on MX3000P Real-time PCR Instrument (Stratagen, US) using Real-time PCR Universal Reagent (GenePharma, Shanghai) according to the manufacturer’s instructions. The relative expression levels of interest gene were calculated by the 2^-ΔΔCt^ method.

### Cell transfection

1×106 cells cultured in a well of 6-well cell culture plate were transiently transfected with 50 pmol of miR-214 inhibitor (or control microRNA) and PTEN siRNA oligonucleotide duplexes (or control siRNA) using Lipofectamine 2000 (Invitrogen, US) according to the manufacturer’s protocol, respectively. The sequence used were: 5’-ACUGCCUGUCUGU GCCUGCUGU-3’ (miR-214 inhibitor oligonucleotide); 5’-CAGUACUUUUGUGUAGUAC AA-3’ (control oligonucleotide). Validated siRNAs directed against PTEN and control siRNA were obtained from Cell Signaling. Transfection efficiency was optimized using 6-carboxyfluorescein labeled microRNA (or siRNA) at approximately 80% in gastric cancer cells.

### Cell viability assay

We used 3-(4,5-dimethylthiazol-2-yl)-2,5-diphenyltetrazolium bromide (MTT) assay to determine the viability of cells. Cells were seeded in 96-well plates at 8000 cells per well. At the end, cells were incubated in 50 ml of 0.1 mg/ml solution of MTT at 37°C for 4 h and then lysed in 150 ml of dimethylsulfoxide at room temperature for 15 min. Viable cell numbers were estimated by measurement of optical density (OD) at 580 nm at various time points.

### Cell population doubling time and cell cycle assay

Cell population doubling time calculation and cell cycle assay were performed as previously described [11]. Cells were seeded into 6-well plates (1 × 10^4^ cells/well) and cultured at 37°C. Cell population doubling time (PDT) was calculated using the following equation: PDT (hr) = (log2×t)/(logN_t_-logN_0_), where t=time in culture (hr), N_t_ = final cell count, N_0_ = original cell count.

In the cell cycle assay, Cells were fixed in 70% ethanol for 2 hr at 4°C, then, the cells were treated with RNaseA (50 μg/ml) and stained with propidium iodide (25 μg/ml) for 30 min at 37°C. Analyzed all samples using an FACSCalibur flow cytometer (BD Biosciences) and distribution of cell-cycle phases was determined using Modfit Software (BD Biosciences). The proliferative index (PI) was calculated as the percentage of cells in S/G2/M-phase.

### Clonogenic assay

Clonogenic assay was also performed as previously described [12]. Single-cell suspension was prepared using trypsin treatment. Cells were then seeded into 6-well cell culture plates (200 cells/well) and incubated for 14 days at 37°C. Then, cells were washed twice with PBS and stained with a mixture of 6.0% glutaraldehyde and 0.5% crystal violet for 1 hour at 37°C. The plates were air dried at room temperature. Colony forming efficiency (CFE) was calculated as the percentage of plated cells that formed colonies.

### Cell migration and invasion assay

A cell suspension of in 0.2 ml RPMI-1640 medium with 5% FBS was seeded into each well of the upper Transwell chamber (8-μm pore size, Corning Costar Corp, US), which was pre-coated with or without Matrigel. In the lower chamber, 0.6 ml RPMI 1640 with 20% FBS was added. After incubating for 28 h 37°C in a humidified incubator with 5% CO_2_, chambers were disassembled and the membranes were stained with 2% crystal violet for 10 min and placed on a glass slide. The number of cells penetrating across membrane were counted under a microscope in ten random visual fields.

### Luciferase reporter assay

Dual-luciferase activity assays were performed as previously described [7]. The full-length 3’-UTR segments of PTEN mRNA containing the miR-214 binding site was amplified by PCR and inserted into the Xba1-site of pGL3 vector (Promega, WI) and named pGL3-PTEN. The pGL3-PTEN-mut reporter construct with point mutations in the seed sequence was synthesized using a site-directed mutagenesis kit (Stratagene, CA). Then, 1 × 10^6^ cells were cotransfected with 50 pmol of miR-214 inhibitor (or control miRNA), 1 μg of pGL3-PTEN (or pGL3-PTEN-mut) plasmid, and 1 μg of a Renilla luciferase expression construct pRL-TK (Promega, WI) using Lipofectamine 2000. After 36 h transfection, luciferase activity was measured using the dual luciferase assay system (Promega, WI) and normalized to Renilla luciferase activity.

### Western blotting

Cells were washed twice with Hanks’s balanced salt solution and lysed directly in lysis buffer (50 mM Tris–HCl, pH 8.0, 1% NP-40, 10 mM NaCl, 2 mM EDTA, 5 mg/ml leupeptin, 2 mg/ml aprotinin, 2 mg/ml pepstatin, 1 mM DTT, 0.1% SDS and 1 mM phenylmethylsulfonyl fluoride). The protein concentrations of the lysates were measured using a Bradford protein assay kit (Bio-Rad, US). Equivalent amounts of protein were separated by 10% SDS PAGE and then transferred to nitrocellulose membranes by electroblotting. The membranes were blocked with 5% BSA in TBST (10 mM Tris–HCl, pH 8.0, 150 mM NaCl, and 0.05% Tween 20) for 1 hr, and then the membrane was immunoblotted overnight at 4°C with primary antibody, a secondary antibody conjugated with horseradish peroxidase was incubated with the membrane for 1 hr at 37°C. Protein was visualized using enhanced chemiluminescence reagent (Santa Cruz). The expression level of PTEN protein was analyzed using LabWork 4.0 program (UVP) and normalized to that of β-actin protein.

### Statistical analysis

Data are presented as mean ± SEM from at least three independent experiments. Statistical significance was analyzed using SPSS11.0 software package (SPSS Inc., US). The difference between groups was performed with Student’s *t*-test. Pearson’s correlation was used to estimate the relationship between expression levels of miR-214 and PTEN mRNA. Survival curves were obtained by the Kaplan-Meier method, comparison between curves was calculated by Log-rank test. Differences were considered significant for p-values less than 0.05.

## Results

### The expression level of miR-214 is up-regulated and inversely correlated with PTEN mRNA in gastric cancer tissues

**Figure 1:**
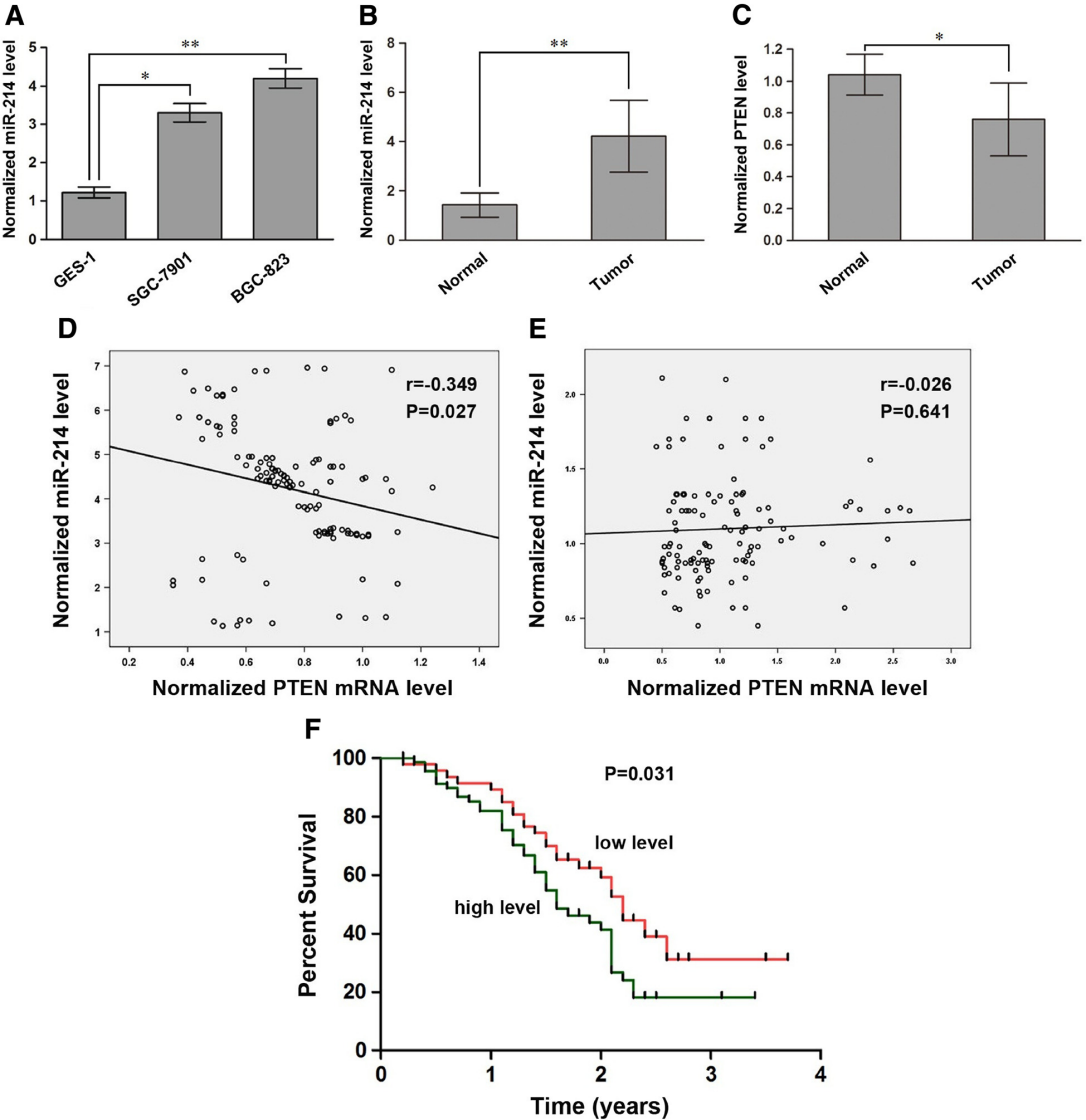
**Expression of miR-214 and PTEN in gastric cancer cell and tissue specimens.** Detection of miR-214 and PTEN mRNA by qRT-PCR using U6 snRNA for normalization. Both SGC-7901 and BGC-823 cells express higher levels of miR-214 compared with normal gastric mucosa epithelial cell lines GES-1 **(A)**, the expression of miR-214 in gastric cancer tissues is higher than that in normal gastric mucosa tissues **(B)**. Whereas, the expression of PTEN mRNA in gastric cancer tissues is lower than that in normal gastric mucosa tissues **(C)**, in addition, we found an inverse correlation between the expression of miR-214 and the level of PTEN mRNA in gastric cancer tissues, but not in normal tissues **(D**, **E)**. Kaplan-Meier survival curves for 120 gastric cancer cases, low expression of miR-214 (red) was defined as long survival, and high expression (green) was defined as short survival **(F)**. *P < 0.05; **P < 0.01.

The quantitative RT-PCR detection results showed that the expression levels of miR-214 were significantly higher in gastric cancer cell lines in comparison with the normal gastric mucosa epithelial cell lines (Figure 1A). In the meantime, miR-214 overexpression is also observed in gastric cancer tissues compared to normal gastric mucosa tissues (Figure 1B). Furthermore, the expression of miR-214 is significantly associated with invasion, metastasis and TNM stage according to the clinicopathologic data from the gastric cancer patients (Table 1), and clinical relevance was confirmed by the observation that miR-214 overexpression correlated with poor prognosis (Figure 1F). All these evidence indicated that miR-214 may be involved in gastric cancer carcinogenesis.

**Table 1:** Association of PTEN mRNA or miR-214 expression with clinicopathological data from gastric cancer patients by quantitative PCR

**Variable**	**N**	**miR-214**		**PTEN**	
		**Relative expression level**	**p-value**	**Relative mRNA level**	**p-value**
Age (years)					
≤ 60	47	3.429±1.924	0.117	0.374±0.238	0.717
> 60	73	4.384±1.317		0.769±0.225	
Tumor size					
≤ 4 cm	67	3.954±1.499	0.101	0.809±0.229	0.060
> 4 cm	53	4.762±1.252		0.667±0.187	
Borrmann’s Classification					
I-II	65	4.168±1.231	0.789	0.748±0.230	0.608
III-IV	55	4.298±1.821		0.787±0.219	
Lauren’s Classification					
Intestinal	64	3.984±1.579	0.416	0.724±0.261	0.377
Diffuse	56	4.372±1.385		0.789±0.198	
Invasive depth					
T_1_-T_2_	52	2.927±1.749	0.001^*^	0.826±0.301	0.310
T_3_-T_4_	68	4.647±1.070		0.741±0.195	
Metastasis					
Negative	76	4.006±1.359	0.005^*^	0.788±0.219	0.031^*^
Positive	44	6.105±0.913		0.535±0.122	
TNM staging					
I-II	57	3.367±1.361	0.001^*^	0.843±0.215	0.047^*^
III-IV	63	4.845±1.205		0.704±0.163	

^*^significantly different.

We also examined the expression levels of PTEN mRNA in gastric cancer tissues and normal gastric mucosa tissues. Our results showed that decreased expression of PTEN mRNA was found in gastric cancer tissues (Figure 1C). Furthermore, we found that PTEN mRNA expression was significantly associated with metastasis and TNM stage (Table 1). Across all specimens tested, we found an inverse correlation between the expression of miR-214 and the level of PTEN mRNA (Figure 1D, r=−0.349, P=0.027), but not in normal tissues (Figure 1E, r=−0.026, P=0.641). These data suggest that miR-214 may be involved in the regulation of PTEN in gastric cancer.

### Knockdown of miR-214 could inhibit the proliferation, migration and invasion of gastric cancer cell lines

**Figure 2:**
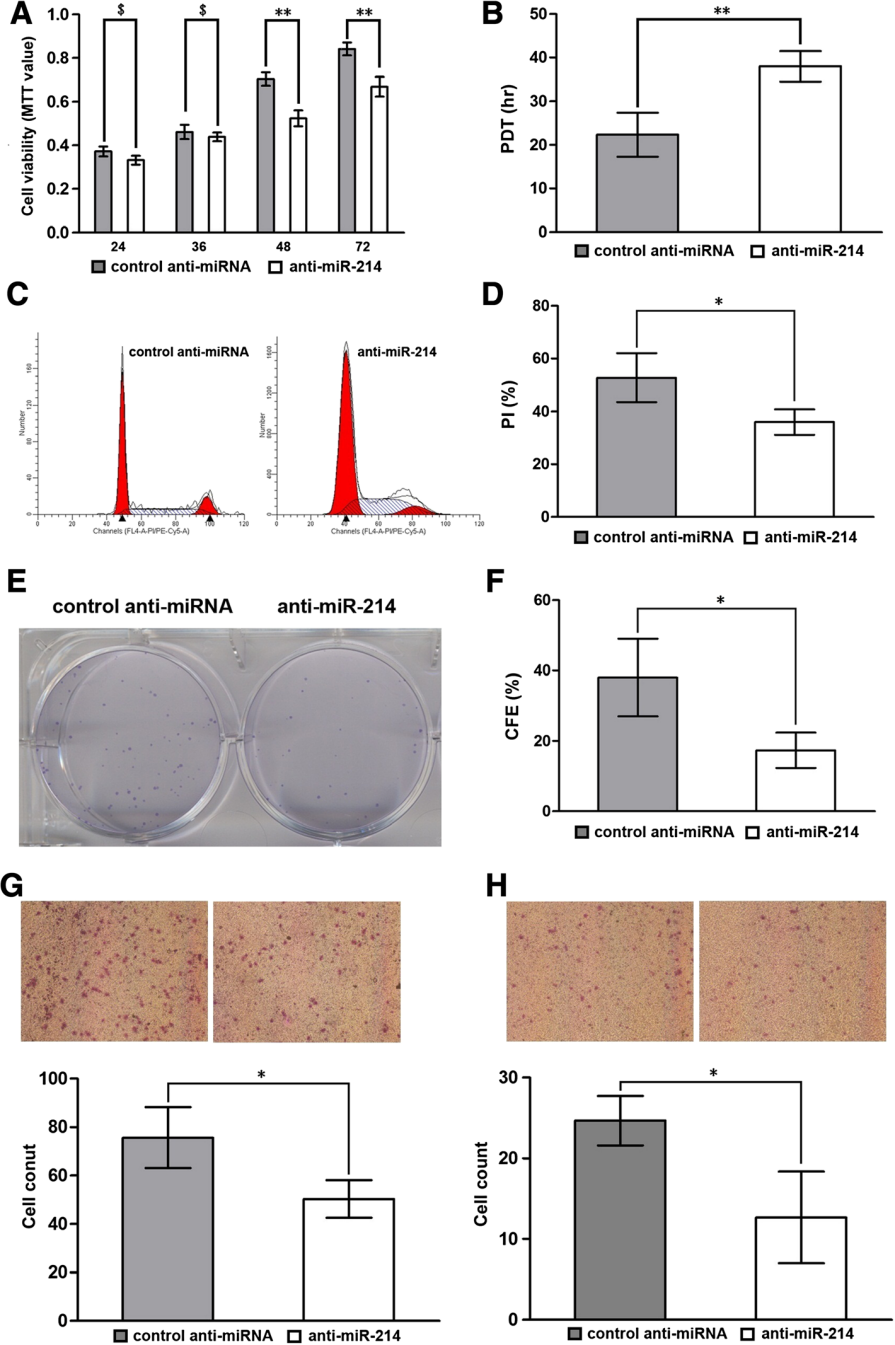
**Down-regulation of miR-214 inhibits the proliferation, migration and invasion of BGC-823 gastric cancer cell lines. (A)** Cell viability was measured at 24 hr post-transfection by MTT assay. Results showed that MTT value of cells transfected with anti-miR-214 was significantly lower than that of cells transfected with control anti-miRNA after 48 hr post-transfection. **(B)** Cell population doubling time (PDT) was determined as described in materials and methods with cells at 24 hr post-transfection. Results showed that the PDT of cells transfected with anti-miR-214 was significantly longer than that of cells transfected with control anti-miRNA. **(C**, **D)** Cell cycle analysis was performed at 48 hr post-transfection by staining DNA with propidium iodide prior to flow cytometry. Results showed that proliferative index (PI) of cells transfected with anti-miR-214 was significantly lower than that of cells transfected with control anti-miRNA. **(E**, **F)** Clonogenic assay was performed as described in materials and methods with cells at 24 hr post-transfection. Results showed that the colony forming efficiency (CFE) of cells transfected with anti-miR-214 was significantly lower than that of cells transfected with control anti-miRNA. **(G**, **H)** Gastric cancer cells transfected with anti-miR-214 and control anti-miRNA were seeded into the upper part of a transwell chamber with or without matrigel, cells migrating across the membrane were counted in all fields. Results showed that number migrating across the membrane with or without matrigel of cells transfected with anti-miR-214 was significantly less than that of cells transfected with control anti-miRNA. Data represent mean ± SEM from three independent experiments; *P < 0.05 by *t* test, **P < 0.01 by *t* test.

We firstly investigated the effect of miR-214 on proliferation of BGC-823 gastric cancer cell lines using MTT assay. As shown in Figure 2A, MTT value of cells transfected with anti-miR-214 was significantly lower than that of cells transfected with control anti-miRNA after 48 hr post-transfection. In addition, cells treated with anti-miR-214 had a significantly longer population doubling time, lower proliferative index and colony forming efficiency than cells transfected with control anti-miRNA (Figure 2B-F). Meanwhile, it showed that cell number migrating across the membrane with or without matrigel of cells transfected with anti-miR-214 was significantly less than that of cells transfected with control anti-miRNA (Figure 2G-H). These results provide strong evidence that knockdown of miR-214 could inhibit the proliferation, migration and invasion of gastric cancer cells.

### miR-214 post-transcriptionally down-regulates PTEN expression by targeting the 3’ untranslated region of PTEN

**Figure 3:**
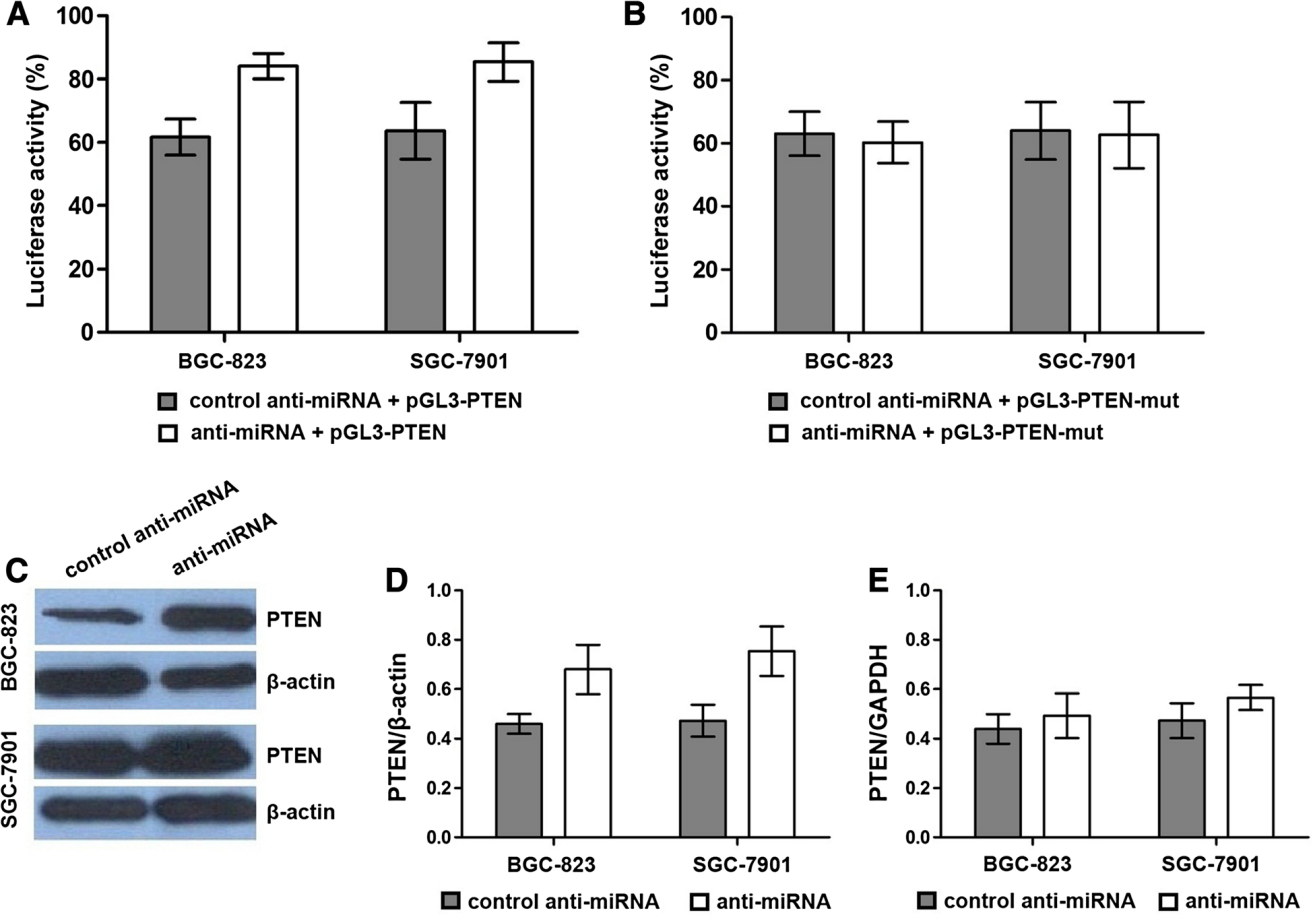
**miR-214 post-transcriptionally regulates PTEN expression by targeting the 3’-UTR of PTEN. (A**, **B)** Luciferase reporter assay were performed at 48 hr post-transfection. BGC-823 and SGC-7901 gastric cancer cell lines were transfected respectively with the Renilla luciferase expression construct pRL-TK and pGL3-PTEN-3’-UTR firefly luciferase expression construct, along with either anti-miR-214 or control anti-miRNA. Results showed that cells co-transfected with miR-214 and pGL3-PTEN plasmid exhibited a significant increase of reporter activity in comparison with those co-transfected with the control anti-miRNA and pGL3-PTEN plasmid. However, the reporter activity of cells co-transfected with anti-miR-214 and pGL3-PTEN-mut plasmid showed no significant difference with that of cells cotransfected with control microRNA and pGL3-PTEN-mut plasmid. **(C**, **D)** The expression level of PTEN protein was detected by Western Blot at 48 hr post-transfection and normalized to that of β-actin. Results showed that the level of PTEN protein was significantly increased in cells transfected with anti-miR-214 as compared to the cells transfected with control anti-miRNA. **(E)** The expression level of PTEN mRNA was detected by qRT-PCR at 48 hr posttransfection and normalized to that of GAPDH. Results showed that the expression level of PTEN mRNA exhibited no significantly difference between cells transfected with anti-miR-214 and those transfected with control anti-miRNA. Data represent mean ± SEM from three independent experiments; *P < 0.05 by *t* test, **P < 0.01 by *t* test.

It was reported that the 3’-UTR of PTEN contains the miR-214 target sequence [13]. To investigate whether miR-214 directly can alter the expression of PTEN in gastric cancer cells, a fragment of the 3’-UTR of PTEN mRNA, containing the putative miR-214 binding sequence, was cloned into a firefly luciferase reporter construct, and cotransfected with a control Renilla luciferase reporter construct into gastric cancer cells along with either anti-miR-214 or control anti-miRNA. As shown in Figure 3A, BGC-823 and SGC-7901 cell lines cotransfected with anti-miR-214 and pGL3-PTEN plasmid showed a significant increase of reporter activity in comparison with those cotransfected with the control anti-miRNA and pGL3-PTEN plasmid. However, cells cotransfected with anti-miR-214 and pGL3-PTEN-mut plasmid showed no significant difference in reporter activity as compared with cells cotransfected with control anti-miRNA and pGL3-PTEN-mut plasmid (Figure 3B).

We further determined the expression of PTEN protein and mRNA by Western blotting and qRT-PCR in gastric cancer cells transfected with anti-miR-214 (or control anti-miRNA). As shown in Figure 3C and 3D, the expression of PTEN protein was significantly increased in cells transfected with anti-miR-214 as compared to the cells treated with control anti-miRNA at 48 hr post-transfection. However, the expression of PTEN mRNA showed no significant difference between the two groups (Figure 3E). These results indicate that the 3’-UTR of PTEN mRNA is a functional target of miR-214 in gastric cancer cells.

### Down-regulation of PTEN could significantly attenuated the inhibitory effects of anti-miR-214 on the proliferation, migration and invasion of gastric cancer cell lines

**Figure 4:**
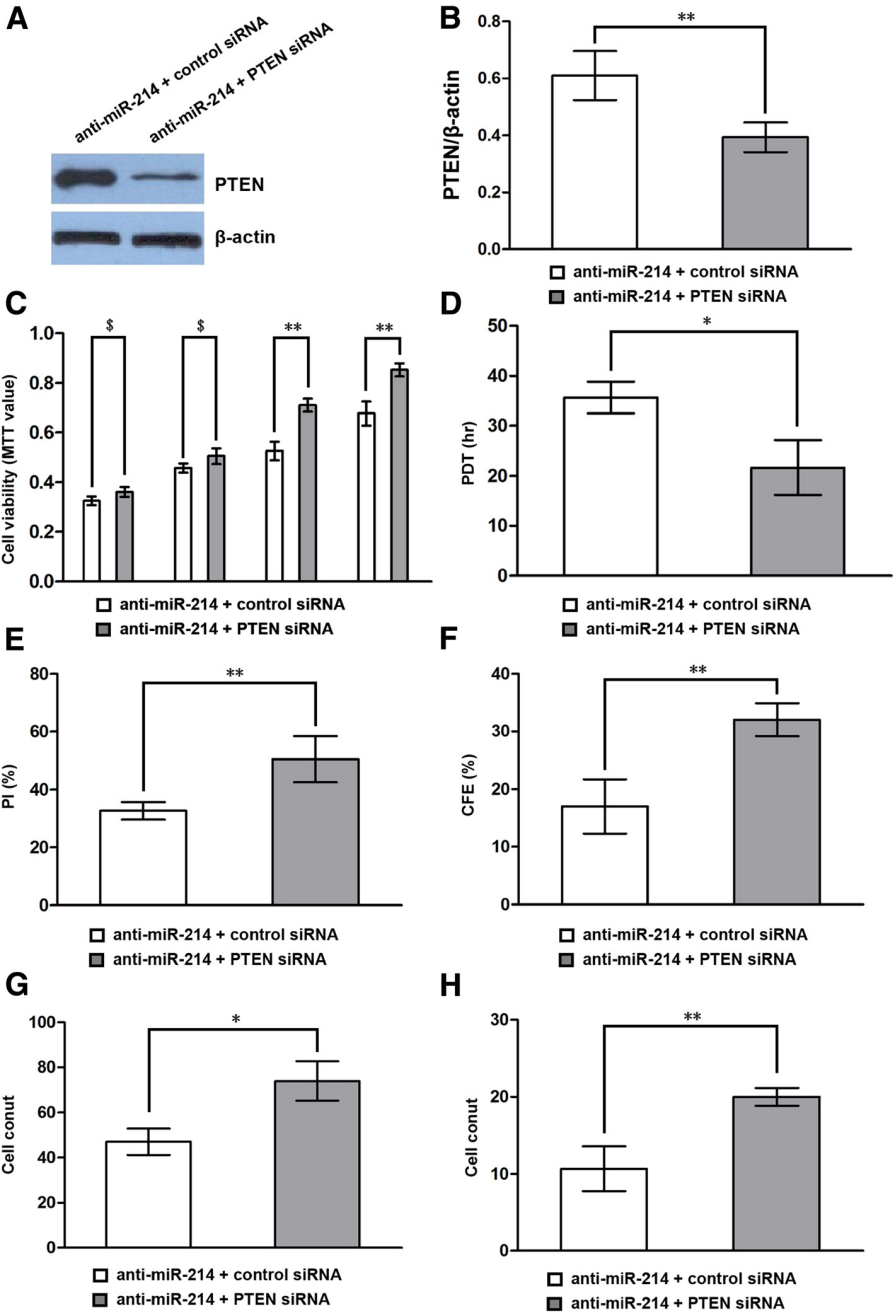
**Down-regulation of PTEN attenuates the effects of anti-miR-214 on BGC-823 gastric cancer cell proliferation, migration and invasion. (A**, **B)** The expression level of PTEN protein was detected by Western-blot at 48 hr post-transfection and normalized to that of β-actin. Results showed that the expression level of PTEN protein was significantly lower in cells co-transfected with anti-miR-214 and siRNA-PTEN as compared to the cells transfected with anti-miR-214 and control siRNA. Cell vability was detected by MTT assay **(C)**, population doubling time determination **(D)**, proliferative index **(E)**, clonogenic assay **(F)**, migration and invasion assay **(G**, **H)** as described in materials and methods. Results showed that the proliferative capacity of cells co-transfected with anti-miR-214 and siRNA-PTEN was significantly higher than that of cells co-transfected with anti-miR-214 and control siRNA. Data represent mean ± SEM from three independent experiments; *P < 0.05 by *t* test, **P < 0.01 by *t* test.

To further evaluate the contribution of PTEN to biological effects of miR-214, we assessed the impact of PTEN silencing by RNA interference, and hence PTEN expression on anti-miR-214 dependent cell proliferation, migration and invasion in BGC-823 gastric cancer cell lines. At 48 hr posttransfection, Western blotting revealed that the expression level of PTEN in cells cotransfected with anti-miR-214 and PTEN siRNA plasmid was significantly lower than that in cells cotransfected with anti-miR-214 and control plasmid (Figure 4A and B). Subsequent studies showed that the effect of anti-miR-214 in decreasing both cell proliferation, migration as well as cell invasion was prevented by the presence of siRNA to PTEN in gastric cancer cell lines (Figure 4C-H). These results indicated that down-regulation of PTEN expression could significantly attenuate the inhibitory effect of anti-miR-214 on cell proliferation, migration and invasion, suggesting that the anti-miR-214 inhibits the proliferation, migration and invasion of gastric cancer cells through the PTEN-mediated signal pathway.

## Discussion

Recent several studies shown the dysregulation of some miRNAs in various types of human cancers, and the alteration of miRNAs expression might contribute to human carcinogenesis by regulating multiple types of target genes expression [13-15]. Hence, identification of specific miRNAs and their targets involved in tumorigenesis would provide valuable insight for the diagnosis and therapy of patients with human malignancies. Here, we have demonstrated that knockdown of miR-214 could inhibit proliferation, migration and invasion capacity of gastric cancer cells by negatively regulating tumor suppressor PTEN at the post-transcriptional level via binding to non-coding regions of PTEN. Our study data suggest that miR-214 may be useful as a novel potential therapeutic approach for the treatment of gastric cancer.

It was previously reported that miR-214 is involved in the murine aging process [16], modulates Hedgehog signaling to specify muscle cell fate [17], and induces cell survival and cisplatin resistance by targeting PTEN in human ovarian cancer [7]. In addition, miR-214 expression was elevated in pancreatic cancer tissues compared with matched benign pancreatic tissues, and overexpression of miR-214 could decreased the sensitivity of the pancreatic cancer cells to gemcitabine by targeting ING4 mRNA [18]. Meanwhile, miR-214 is down-regulated in human cervical cancer tissue compared with normal tissue and that it negatively regulates HeLa cell proliferation by targeting the noncoding regions of MEK3 and JNK1 mRNAs [19]. In the present study, we found that the expression level of miR-214 was up-regulated in gastric cancer tissue compared with matched normal tissue, and miR-214 expression level was significantly associated with clinical progression and poor prognosis. Specially, we found that the proliferative, migratory and invasive capacity of gastric cancer cells transfected with anti-miR-214 was significantly lower than that of cells transfected with control anti-miRNA, suggesting that repressing miR-214 expression could inhibit the proliferative and progressive capacity of gastric cancer cells.

PTEN is a protein tyrosine phosphatase and tensin homologue, which was first discovered by independent laboratories and identified as a tumor suppressor gene located on human chromosome region 10q23 [20]. It has been reported that PTEN makes a great contribution to cellular apoptosis, proliferation, migration and invasion, as well as angiogenesis through interference with several signaling pathways [21-24]. Recent studies have shown that PTEN protein expression frequently is decreased or absent in human gastric cancer. Moreover, decreased PTEN expression correlates with differentiation, growth, progression and angiogenesis in gastric cancer [25,26]. In addition, up-regulation of PTEN can increase expression of Caspase-3 to make tumor cells apoptosis disorder, which forms molecular mechanisms of PTEN contribution to tumorigenesis and progression of gastric cancer [27].

In this study, we confirmed that PTEN is down-regulated in gastric cancer in vivo, consistent with the previous findings. Moreover, the expression of PTEN is positively correlated with metastasis and invasion from the clinicopathologic data analysis. In addition, we observed a highly significant negative correlation between miR-214 and PTEN expression in tumor tissues, suggesting that miR-214 could be involved in the regulation of PTEN in gastric cancer. Combining with the target reporter assays, we further have demonstrated that miR-214 post-transcriptionally regulates PTEN via binding the 3’-UTR of PTEN mRNA. Considering the different miRNA have different roles and target genes in different tumors, so we have choosed two gastric cancer cell lines as a model to validated the regular effects of miR-214 on PTEN gene.

In summary, we have demonstrated that miR-214 is overexpressed in gastric cancer, and knockdown of miR-214 can significantly inhibit the proliferation, migration and invasion capacity of gastric cancer cell through the PTEN-mediated signal pathway, which has not been documented in previous studies. Our data suggest that miR-214 is possible to become a potential therapeutic agent for gastric cancer.

## Competing interests

The authors declare that they have no competing interests.

## Authors’ contributions

SZS conceived the design of the study and was in charge of its coordination. TSY participated in data analysis, performed data interpretation and drafted the manuscript. XHY carried out the cell proliferation analysis and helped to draft the manuscript. XDW performed molecular biology experiment and helped to draft the manuscript. YLW participated in cell culture and Luciferase reporter assay. BZ participated in flow cytometry analysis. All authors read and approved the final manuscript.
